# Direct targeting of sEH with alisol B alleviated the apoptosis, inflammation, and oxidative stress in cisplatin-induced acute kidney injury

**DOI:** 10.7150/ijbs.78097

**Published:** 2023-01-01

**Authors:** Juan Zhang, Zhi-Lin Luan, Xiao-Kui Huo, Min Zhang, Christophe Morisseau, Cheng-Peng Sun, Bruce D. Hammock, Xiao-Chi Ma

**Affiliations:** 1 College of Pharmacy, Second Affiliated Hospital, Dalian Medical University, Dalian 116044, China.; 2 School of Pharmaceutical Sciences, Health Science Center, Shenzhen University, Shenzhen 518061, China.; 3 Department of Entomology and Nematology, UC Davis Comprehensive Cancer Center, University of California, Davis, CA 95616, United States.

**Keywords:** Alisol B, acute kidney injury, cisplatin, soluble epoxide hydrolase, nephrotoxicity

## Abstract

Acute kidney injury (AKI) is a pathological condition characterized by a rapid decrease in glomerular filtration rate and nitrogenous waste accumulation during hemodynamic regulation. Alisol B, from *Alisma orientale,* displays anti-tumor, anti-complement, and anti-inflammatory effects. However, its effect and action mechanism on AKI is still unclear. Herein, alisol B significantly attenuated cisplatin (Cis)-induced renal tubular apoptosis through decreasing expressions levels of cleaved-caspase 3 and cleaved-PARP and the ratio of Bax/Bcl-2 depended on the p53 pathway. Alisol B also alleviated Cis-induced inflammatory response (e.g. the increase of ICAM-1, MCP-1, COX-2, iNOS, IL-6, and TNF-α) and oxidative stress (e.g. the decrease of SOD and GSH, the decrease of HO-1, GCLC, GCLM, and NQO-1) through the NF-κB and Nrf2 pathways. In a target fishing experiment, alisol B bound to soluble epoxide hydrolase (sEH) as a direct cellular target through the hydrogen bond with Gln384, which was further supported by inhibition kinetics and surface plasmon resonance (equilibrium dissociation constant, *K*_D_ = 1.32 μM). Notably, alisol B enhanced levels of epoxyeicosatrienoic acids and decreased levels of dihydroxyeicosatrienoic acids, indicating that alisol B reduced the sEH activity *in vivo*. In addition, sEH genetic deletion alleviated Cis-induced AKI and abolished the protective effect of alisol B in Cis-induced AKI as well. These findings indicated that alisol B targeted sEH to alleviate Cis-induced AKI *via* GSK3β-mediated p53, NF-κB, and Nrf2 signaling pathways and could be used as a potential therapeutic agent in the treatment of AKI.

## Introduction

*cis*-Diamminedichloroplatinum (II) (cisplatin, Cis) is a chemotherapeutic drug to be used for the treatment of solid tumors due to its efficacy in slowing cancer growth 1-5. Its clinical application is frequently complicated as significant nephrotoxicity, ototoxicity, and neurotoxicity 1, 2. 50-100 mg/m^2^ of Cis dose induces nephrotoxicity in approximately one-third of the patients, resulting in the occurrence of acute kidney injury (AKI) 6, 7. As the most common reason for renal consultation, AKI is a pathological condition with the characteristics of the nitrogenous waste accumulation and the rapid decrease of glomerular filtration rate during hemodynamic regulation 8. The continuously growing incidence of AKI is a global health concern for the scientific community 9. Although its mechanism is still limited, some factors, such as cell apoptosis, inflammation, and oxidative stress, are associated with Cis-induced AKI 10. Cis usually accumulates in renal proximal tubule segment of nephron, and cross-links genomic DNA to distort the duplex structure and stall the replication fork, triggering the activation of the p53 signaling pathway to induce cell apoptosis 1, 2, 11-14. Furthermore, Cis exposure results in the inflammatory response to release a mass of proinflammatory cytokines, contributing to the development of renal tissue damage and renal failure 1, 2, 15. Additionally, accumulating Cis in renal tubule induces oxidative stress to produce reactive oxygen species (ROS), such as superoxide anion and the hydroxyl radical, resulting in the exacerbation of Cis-stimulated apoptosis and inflammation 1, 2, 16. Therefore, regulating apoptosis, inflammation, and oxidative stress serves as a preferred strategy for attenuating Cis-induced AKI.

Polyunsaturated fatty acids (PUFAs) belong to the number of the ω-3 and ω-6 families, and function as a vital role for maintaining the physiological health 17-19. The best studied of PUFAs arachidonic acid (AA), locates in membranes as the form of phospholipids, is metabolized by cytochrome P450s (CYPs) into bioactive derivatives 20. As the most representative metabolite, epoxyeicosatrienoic acids (EETs), from AA produced by CYP450 oxidases, have received a great attention from scientists due to their physiological effects 20-22. However, soluble epoxide hydrolase (sEH) is able to rapidly metabolize EETs, causing the loss of their multiple effects 18, 23-28. sEH (genetic name *Ephx2*) is widely distributed in tissues, particularly kidney and liver 29, 30. Its C-terminal mediates the hydrolysis of bioactive epoxy fatty acids (EpFAs), such as EETs 20, 31, while the role of the N-terminal phosphatase is poorly understood. Recently, the expression and activity of sEH have been reported in connection with kidney diseases 32, and *Ephx2* genetic deletion and inhibition of sEH both attenuate renal diseases 32-34. For example, sEH inhibitors, AUDA and AR9273, significantly attenuated the renal functions (e.g. creatinine (Cr) and blood urea nitrogen (BUN)) in Cis-induced AKI 29, 34, 35. Therefore, sEH inhibition to enhance the level of EETs has become an attractive strategy to treat diseases related to inflammation, such as AKI and lung injury 36, 37.

The effects of natural medicines in the prevention and treatment of kidney diseases, such as AKI and CKD, are receiving increasing attention in recent years 38.* Alismatis Rhizoma* has been widely and historically used in clinical practice for the treatment of dysuria, edema, diarrhea, hypertension, hyperlipidemia, and nephropathy 39, 40. Alisol B possesses a four-ring protostane skeleton with a side chain at C-17, and is the main constituent of *Alismatis Rhizoma* which only is known to exist in the genus* Alisma* and be served as characteristic markers of this genus 41-43. Pharmacological studies focused on alisol B have revealed its multiple biological effects, including anti-complement, anti-allergy, anti-inflammation, and others 44. A study by Zhao et al. demonstrated that alisol B could alleviate non-alcoholic steatohepatitis via reducing the accumulation of hepatocyte lipid, inflammation, and lipotoxicity in the animal model 45. Furthermore, its analogue alisol B 23-acetate alleviated the course of AKI and chronic kidney disease (CKD) 43, 46, and the network pharmacology study focused on diabetic nephropathy indicated the reno-protective potential of alisol B 47. Herein, we investigated beneficial effects of alisol B toward renal tubular apoptosis, inflammatory response, and oxidative stress in the Cis-induced AKI mouse model, and revealed its drug target and underlying mechanism.

## Materials and Methods

### Chemicals and reagents

Alisol B was isolated from *A. orientale* by authors, and identified through ^1^H and ^13^C NMR spectra. Recombinant human sEH was purchased from Cayman Chemical and used to detect the *K*_D_ value of alisol B with sEH. Hematoxylin and eosin (H&E) staining kit was obtained from Beyotime (Shanghai, China). The primary antibodies were purchased from Proteintech (Wuhan, China), Cell Signaling Technology (CST, Danvers, MA, USA), Abcam (Cambridge, United Kingdom), Boster (Wuhan, China) and Abclonal (Wuhan, China). Detailed information for the primary antibodies were as following: p53 (10442-1-AP, Proteintech), PARP (9542S, CST), Bcl-2 (3498S, CST), Bax (ab182734, Abcam), Caspase3 (ab49822, Abcam), Cleave-Caspase3 (9664S, CST), β-actin (BM0627, Boster), MCP-1 (66272-1-lg, Proteintech), ICAM-1 (60299-1-lg, Proteintech), iNOS (13120S, CST), IL-6 (21865-1-AP, Proteintech), p65 (8242T, CST), Grp78 (ab21685, abcam), 4-HNE (ab48506, Abcam), TNF-α (60291-1-lg, Proteintech), 8-OXO (ab206461, Abcam), HO-1 (A1346, ABclonal), Nrf2 (ab31163, Abcam), Keap1 (10503-2-AP, Proteintech), sEH (10833-1-AP, Proteintech), Kim-1 (14971S, CST), GSK3β (A2081, ABclonal), and p-GSK3β (5558P, CST).

### Animal models

Wild-type (WT) C57BL/6 mice were obtained from the Experimental Animal Center of Dalian Medical University (Dalian, China), sEH (gene name *Ephx2*, responsible for encoding the sEH protein) gene knockout (KO) mice (21-24 g) were obtained from Cyagen Biosciences Inc. (Suzhou, China), and kept under a light/dark cycle of 12 h per day at a controlled temperature. The genotyping and confirmation of the sEH KO mice are shown in **Figure S5**. All the animal experiments were in line with the Institutional Animal Care and Use Committee of Dalian Medical University (NO. AEE19044).

Male C57BL/6 WT mice (23-25 g) were randomly classified into 6 groups (12/group): the control group, alisol B (60 mg/kg) group, Cis group, Cis + alisol B (15 mg/kg) group, Cis + alisol B (30 mg/kg) group, and Cis + alisol B (60 mg/kg) group. Mice were pretreated with alisol B (15, 30, or 60 mg/kg, dissolved in 10% hydroxypropyl β-cyclodextrin) via oral gavage for seven days. On Day 5, mice in Cis and Cis + alisol B groups were subjected (i.p.) to Cis (20 mg/kg). Mice were sacrificed on Day 8, and the blood and kidneys were collected. The mice in control group were administrated with the vehicle (10% hydroxypropyl β-cyclodextrin and saline).

### Measurement of plasma and kidney

The plasma and kidney samples were prepared and stored at -80 °C after the mice were sacrificed. Cr, BUN, malonyldiadehyde (MDA), glutathione (GSH) and superoxide dismutase (SOD) were measured using the corresponding assay kits (Jiancheng Bioengineering Institute, Nanjing, China).

### Renal pathological assessments and immunohistochemical staining

Formalin-fixed and paraffin-embedded renal sections were stained using H&E or periodic acid-Schiff's (PAS) to identify injured tubules according to standard protocols. The acute tubular necrosis (ATN) score was determined on the basis of the previous criteria 48. Immunohistochemical staining were performed in a routine procedure. In brief, renal sections were incubated with 3% H_2_O_2_, blocked with 5% BSA at 37 °C, and incubated with primary antibodies at 4 °C overnight. The sections were treated with a horseradish peroxidase-conjugated secondary antibody at 37 °C. After 30 min, the sections were visualized with diaminobenzidine. Immunohistochemical signals were quantified by Image J software.

### Quantitative real-time PCR

Total RNA was extracted from C57BL/6 mice and sEH KO mice by using TRIzol reagent (Accurate Biotechnology, Changsha, China), and the reverse transcription experiment was performed by using its corresponding kit to afford the cDNA. The PCR experiments were performed on an CFX96 Real-time System (Bio-Rad, California, USA) with MonAmp SYBR Green qPCR SuperMix (Monad, Shanghai, China). All measured values were normalized with β-actin according to the ΔΔCT method. These primers were listed in **Table S1**.

### Western blot

Kidney samples were extracted with RIPA Lysis buffer with the cocktail, and then centrifugated at 4 °C for 15 min to afford the supernatants used for the determination of the protein concentrations and Western blot analysis. The proteins were subjected to 10%-12% SDS-PAGE, transferred to polyvinylidene difluoride (PVDF) membranes, blocked with 5% skim milk, and incubated with the primary antibodies overnight at 4 °C. The membranes were subsequently incubated with a horseradish peroxidase-conjugated secondary antibody for 1 h, and detected using the Tanon 5200- ECL detection system. Image J software was used for the quantitative analysis.

### LC-MS analysis for Epoxyeicosatrienoic acids (EETs) and dihydroxyeicosatrienoic acid (DHETs)

The levels of EETs and DHETs were measured as previously described 49-51. Kidney samples were homogenized with water to afford the supernatant after the centrifugation at 20,000 g for the analysis using LC-MS/MS.

### Flow cytometry

Mice were anesthetized and perfused with normal saline to collect the kidneys in the ice plate. The kidney samples were cut into pieces (about 1-2 mm), and washed by PBS for tree times. The samples were digested by the collagenase at 37 °C for 30 min to afford renal cells after the centrifugation. Renal cells were stained by Annexin V and propidium iodide (PI) at 4 °C for 30 min, and then analyzed using the flow cytometer.

### Cell culture and treatment

HK-2 cells were cultured in Dulbecco's modified eagle medium (DMEM) with 10% fetal bovine serum (FBS) at 37 °C in humidified air containing 5% CO_2_ at 37 °C. Cells were seeded into the 6-well plate overnight, and then pretreated with or without alisol B (20 μM) and LiCl (5 mM, a GSK3β inhibitor) for 1 h before the challenge with Cis (20 μM). After 24 h, cells were harvested for PCR and Western blot analyses.

### Target protein identification for alisol B

The protein target of alisol B was identified using a pulled-down assay 52. Epoxy-activated Sepharose 6B beads (GE Healthcare, Chicago, USA) was used for the coupling of alisol B. HEK293 lysates were treated with alisol B-coupled beads or alisol B overnight at 4 °C, the bead-captured proteins were washed with PBS, then the beads were boiled with loading buffer for 10 min. The supernatant was separated by 10% SDS-PAGE, followed by the silver staining, Western blot, and LC-MS/MS analysis.

### Cellular thermal shift assay (CETSA)

HEK293 lysates were incubated with alisol B or the vehicle for 30 min, and then heated at different temperatures for 3 min. After the centrifugation of the heated lysates at 20,000 g for 20 min at 4 °C, the supernatants were analyzed by Western blot.

### Solvent-induced protein precipitation (SIP) assay

HEK293 lysates were incubated with alisol B or the vehicle as our previous method 52, 53. The lysates were incubated with alisol B or the vehicle for 30 min, and then treated with acetone: ethanol: acetic acid (AEA, 50: 50: 0.1) for 20 min at 37 °C. After the centrifugation at 20,000 g for 20 min at 4 °C, the supernatants were analyzed by Western blot.

### Drug affinity responsive target stability (DARTS) assay

Before the treatment with pronase (15 min at room temperature), HEK293 lysates were incubated with alisol B (5, 10, and 20 μM) for 1 h, and then isolated by SDS-PAGE for Western blot detection 52.

### Surface plasmon resonance (SPR) assay

Biacore T200 instruments (GE Healthcare) were used to analyze the binding affinity of alisol B with sEH as previously described 51.

### Soluble epoxide hydrolase activity assessment

The inhibitory activity of alisol B towards sEH was also assayed as previously described 54, 55. The sEH (0.1 ng/mL) was pre-incubated with alisol B for 3 min, and then added the probe PHOME (10 μM). After 20 min incubation at 37 °C, the fluorescence signal was recorded on a microplate reader.

### Molecular dynamics simulation

The interaction of alisol B with sEH (PDB: 4OCZ) was analyzed using GROMACS package as previous methods 56, 57.

### Statistical analysis

Statistical analysis was carried out using one-way or two-way ANOVA with the Prism software package. Data were presented as means ± standard error of the mean (SEM). Results were considered significant at *p*<0.05.

## Results

### Alisol B ameliorated Cis-induced AKI in mice

**Figure 1:**
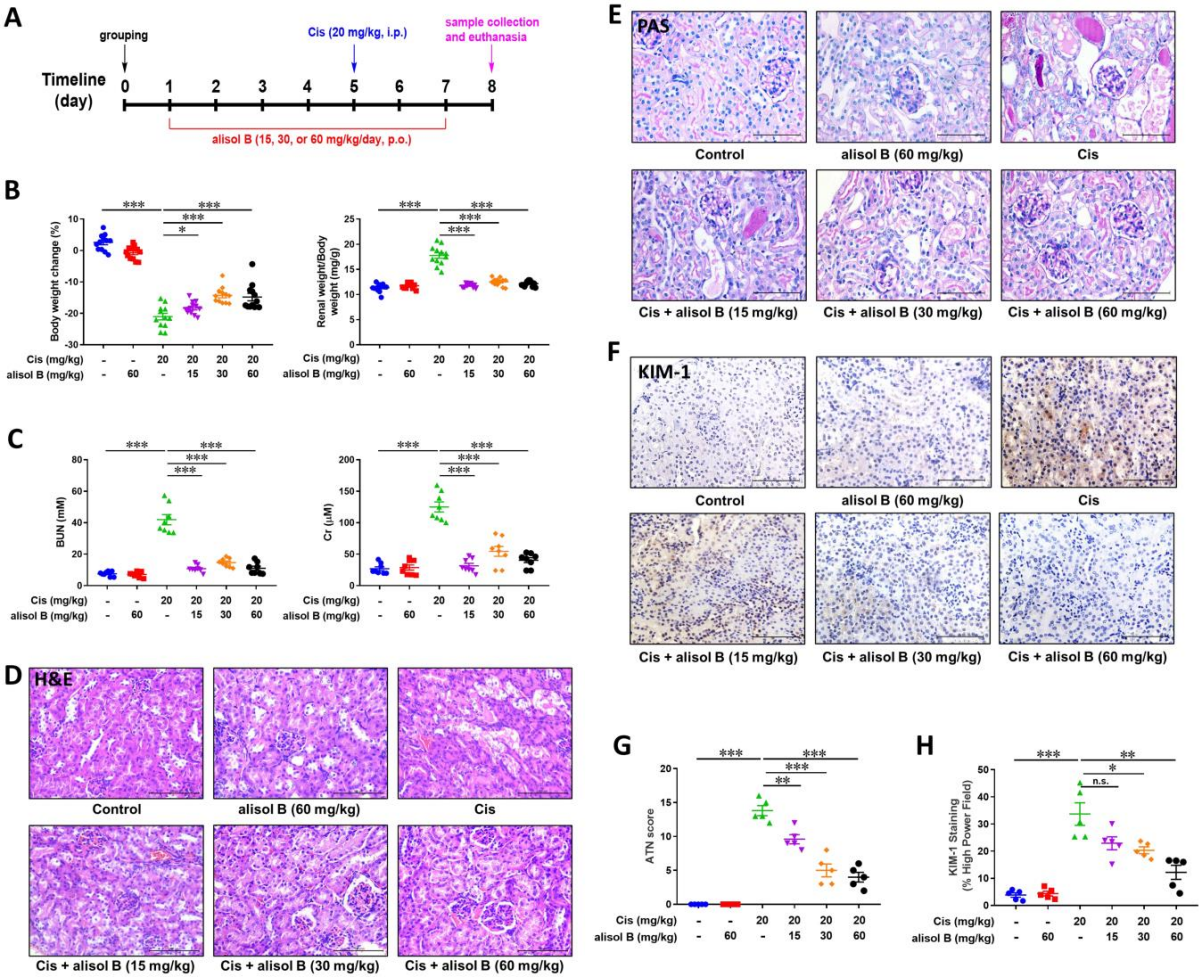
** Alisol B ameliorated Cis-induced AKI in mice. (A)** Schematic diagram of Cis-induced AKI with alisol B treatment. Mice were intragastrically administrated with alisol B (15, 30, or 60 mg/kg/day) daily for 7 days and intravenously treated with Cis (20 mg/kg) on day 4. The animals were sacrificed on day 8; **(B)** Body weight and kidney weight of mice were measured daily; data were presented as mean ± SEM, n=12, *p<0.05, ***p<0.001; **(C)** Measurement of blood urea nitrogen (BUN) and serum creatinine (Cr); data were presented as mean ± SEM, n=8, *** p<0.001; **(D)** Representative images showing H&E staining on renal sections from mice in different groups; **(E)** Representative images showing PAS staining on renal sections from mice in different groups; **(F)** Immunostaining of kidney injury molecule 1 (KIM-1), a specific renal injury marker, on renal sections from mice in different groups. **(G)** ATN scoring of histopathological features in different groups of mice; data were presented as mean ± SEM, n=5, **p<0.01, ***p<0.001; **(H)** Quantitative analysis of KIM-1 positive staining in different groups; data were presented as mean ± SEM, n=5, *p<0.05, ** p<0.01, ***p<0.001.

In order to investigate the effect of alisol B in Cis-induced AKI, mice were treated with alisol B (15, 30, and 60 mg/kg) and then treated with Cis (20 mg/kg) as described in **Figure 1A**. Cis (20 mg/kg) significantly decreased mouse body weight and increased the mouse renal weight/body weight, whereas these changes were reversed by alisol B (15, 30, and 60 mg/kg) in a dose-dependent manner (**Figure 1B&S1**). Furthermore, Cis (20 mg/kg) treatment resulted in renal dysfunction, such as elevated levels of BUN and Cr and obvious renal histological damage (**Figure 1C-E**). Alisol B administration markedly improved renal function impacted by Cis. The increase of BUN and creatinine induced by Cis was dose-dependently attenuated by alisol B (15, 30, and 60 mg/kg) (**Figure 1C**). The results of H&E and PAS staining revealed that Cis (20 mg/kg) treatment led to the extensive necrosis of the proximal tubules compared with the control group (**Figure 1D, E, & G**). However, the mice in control and alisol B-treated groups exhibited relatively normal morphology with alleviated renal tubule damage (15, 30, and 60 mg/kg) (**Figure 1D, E, & G**), which was further supported by the result of of the kidney injury molecule 1 (KIM-1) staining (**Figure 1F&H**). These results suggested that alisol B protected against Cis-induced AKI.

### Alisol B attenuated Cis-induced renal cell apoptosis through p53 signaling pathway

**Figure 2:**
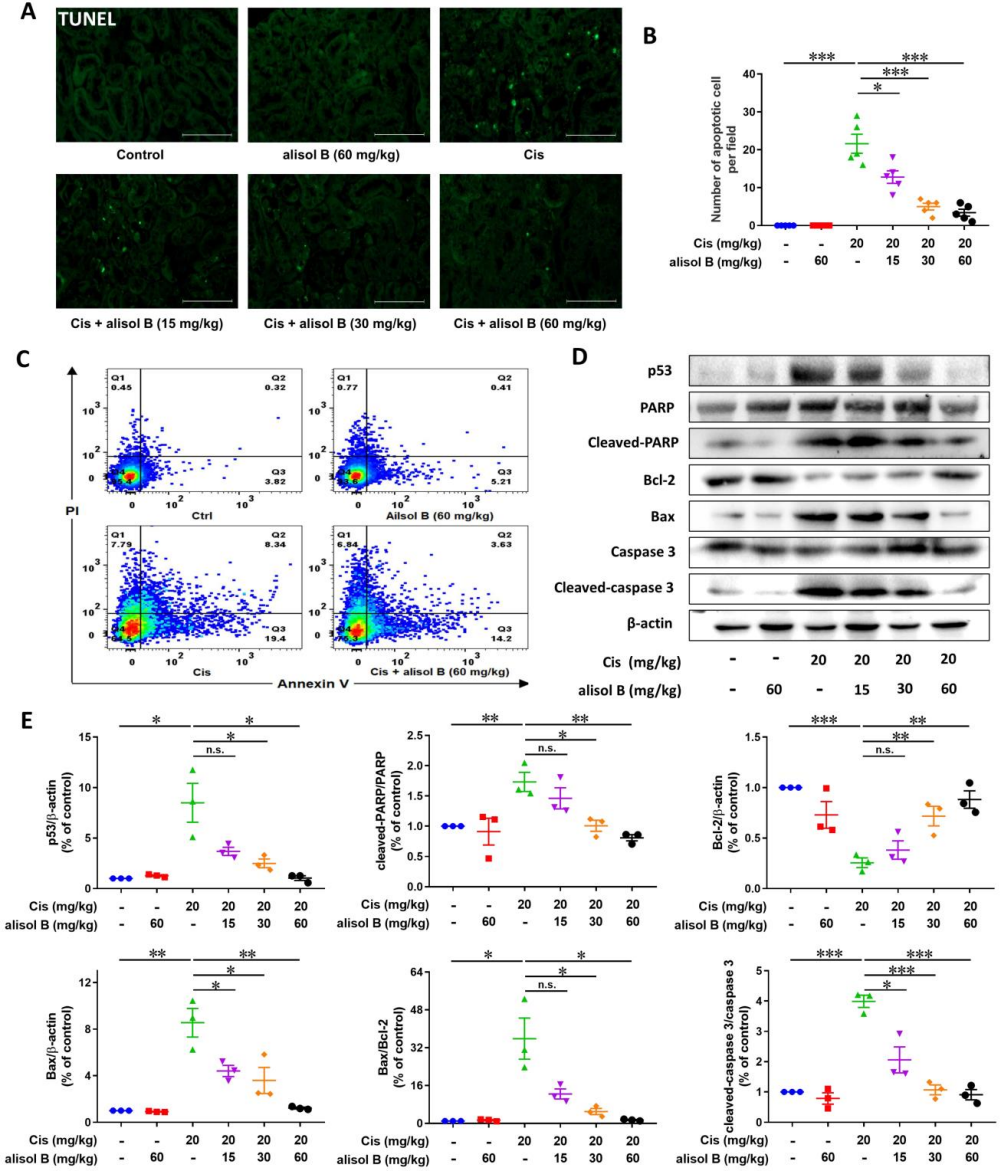
** Alisol B inhibited renal cell apoptosis induced by Cis in mice. (A)** Representative images of TUNEL staining on the renal sections from mice in different groups; **(B)** Quantitative analysis of TUNEL staining; data were presented as mean ± SEM, n=5, *p<0.05, ***p<0.001; **(C)** Representative images of Annexin-V/PI staining on the renal cells from mice in different groups analyzed by flow cytometry; **(D)** Western blot showing the effect of alisol B on protein levels of genes important in cell apoptosis after Cis challenge; **(E)** Quantitative analysis for the protein levels in (**D**); data were presented as mean ± SEM, n=3, *p<0.05, ** p<0.01, ***p<0.001, n.s.= no significance.

Cis treatment resulted in significant cell apoptosis in the kidneys, while alisol B (15, 30, and 60 mg/kg) reduced the number of TUNEL-positive nuclei (**Figure 2A&B**), which was further supported by the flow cytometry experiment through the Annexin-V/PI staining showing that alisol B remarkably attenuated Cis-induced renal cell apoptosis (**Figure 2C&S2**). In parallel, Western blot analyses showed that alisol B (15, 30, and 60 mg/kg) reversed the increase of expression levels of cleaved-caspase 3, cleaved-PARP, p53, and the ratio of Bax/Bcl-2 (**Figure 2D&E**). Therefore, alisol B exerted anti-apoptotic effect through p53 pathway in Cis-induced AKI mice.

### Alisol B alleviated Cis-induced renal inflammation through NF-κB signaling pathway

**Figure 3:**
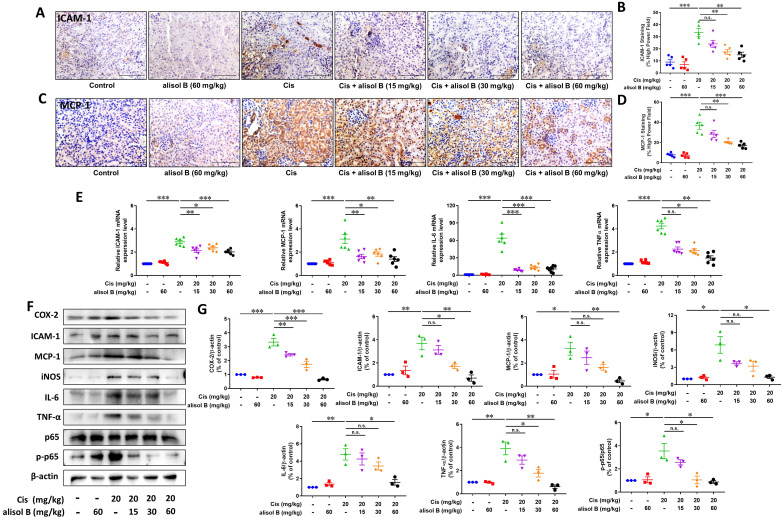
** Alisol B reduced inflammation in the kidneys of Cis-treated mice. (A)** Immunostaining of intracellular adhesion molecule-1 (ICAM-1) on renal sections from mice in different groups;** (B)** Quantitative analysis of ICAM-1 positive staining in different groups; data were presented as mean ± SEM, n=5, ** p<0.01, ***p<0.001, n.s.= no significance;** (C)** Immunostaining of monocyte chemoattractant protein-1 (MCP-1) on renal sections from mice in different groups;** (D)** Quantitative analysis of MCP-1 positive staining in different groups; data were presented as mean ± SEM, n=5 per group, * p<0.05, ***p<0.001; **(E)** qPCR analysis showing the mRNA levels of inflammation-related genes, ICAM-1, MCP-1, IL-6 and TNF-α, in mouse kidneys from different groups; data were presented as mean ± SEM, n=6, *p<0.05, ** p<0.01, ***p<0.001, n.s.= no significance;** (F)** Western blot demonstrating the effect of alisol B on expression levels of COX-2, ICAM-1, MCP-1, iNOS, IL-6, TNF-α, p-p65, and p65 after Cis challenge; **(G)** Quantitative analysis for the protein levels in **(F)**; data were presented as mean ± SEM, n=3, *p<0.05, ** p<0.01, n.s.= no significance.

Cis treatment results in the upregulation of various proinflammatory cytokines and chemokines in the kidneys. To examine the effect of alisol B against renal inflammation on the basis of nuclear factor-kappa B (NF-κB) signaling pathway, we determined expression levels of NF-κB p-p65 and its downstream target proteins and genes, such as inducible nitric oxide synthase (iNOS), intracellular adhesion molecule-1 (ICAM-1), interleukin-6 (IL-6), monocyte chemoattractant protein-1 (MCP-1), tumor necrosis factor-alpha (TNF-α), and cyclooxygenase-2 (COX-2). As shown in **Figure 3A-D**, renal sections from Cis-treated mice displayed significantly increased staining of ICAM-1 and MCP-1, while alisol B (15, 30, and 60 mg/kg) markedly reduced staining of ICAM-1 and MCP-1 induced by Cis (**Figure 3A-D**). Consistent with the immunostaining results, alisol B (15, 30, and 60 mg/kg) dose-dependently regulated protein and mRNA expression levels of MCP-1 and ICAM-1 in Cis-induced mice (**Figure 3E-G**). Cis treatment resulted in the increase of the phosphorylated p65 expression level and its downstream target proteins and genes IL-6, iNOS, TNF-α, and COX-2, whereas their expression levels were dose-dependently decreased in Cis-induced mice *via* alisol B (15, 30, and 60 mg/kg, **Figure 3E-G**). Collectively, alisol B protected Cis-induced inflammation *via* NF-κB signaling pathway.

### Alisol B alleviated Cis-induced renal oxidative and endoplasmic reticulum stresses through Nrf2 signaling pathway

**Figure 4:**
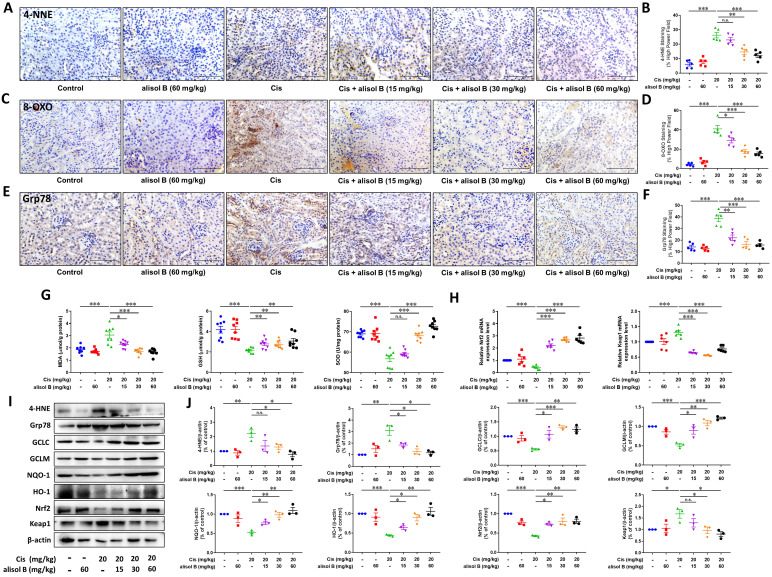
** Alisol B decreased oxidative stress and ER stress in Cis-induced AKI. (A-F)** Representative images and quantitative analysis of immunostaining for 4-HNE **(A&B)**, 8-OXO** (C&D)**, and Grp78 **(E&F)** on renal sections from mice in different groups; data were presented as mean ± SEM, n=5 per group, * p<0.05, ** p<0.01, ***p<0.001, n.s.= no significance;** (G)** Measurement of renal MDA, GSH, and SOD from mice in different groups; data were presented as mean ± SEM, n=8, *p<0.05, ** p<0.01, ***p<0.001, n.s.= no significance; **(H)** qPCR analysis showing the mRNA levels of oxidative stress-related genes, Nrf2 and Keap1, in mouse kidneys from different groups; data were presented as mean ± SEM, n=6 per group, ***p<0.001; **(I)** Western blot demonstrating the effect of alisol B on expression levels of 4-HNE, Grp78, GCLC, GCLM, NQO-1, HO-1, Nrf2, and Keap1 after Cis challenge; **(J)** Quantitative analysis for the protein levels in **(I)**; data were presented as mean ± SEM, n=3, *p<0.05, ** p<0.01, *** p<0.001, n.s.= no significance.

Oxidative and endoplasmic reticulum (ER) stresses are the important pathological course of AKI, therefore, we investigated whether alisol B protected against Cis-induced AKI by attenuating oxidative and ER stresses. We firstly examined the immunostaining of markers for reactive oxygen species (ROS) production and ER stress, 8-oxo-2′-deoxyguanosine (8-OXO), glucose-regulated protein 78 (Grp78), and 4-hydroxynonenal (4-HNE). The immunostaining results showed that alisol B (15, 30, and 60 mg/kg) markedly reduced Cis-induced expressions of 4-HNE, 8-OXO, and Grp78 (**Figure 4A-F**). Furthermore, alisol B (15, 30, and 60 mg/kg) reduced the MDA level, and enhanced GSH and SOD levels in Cis-induced AKI mice (**Figure 4G**). As a key transcription factor, nuclear factor erythroid 2-related factor 2 (Nrf2) is in charge of oxidative and ER stresses, we found that alisol B (15, 30, and 60 mg/kg) could activate Nrf2 signaling pathway *via* upregulating expression levels of HO-1, GCLC, GCLM, NQO-1, and Nrf2, and downregulating Keap1 mRNA and expression levels (**Figure 4H-J**) in Cis-induced AKI mice. These findings indicated that alisol B alleviated Cis-induced oxidative and ER stresses *via* Nrf2 signaling pathway.

### sEH as a direct cellular target of alisol B

**Figure 5:**
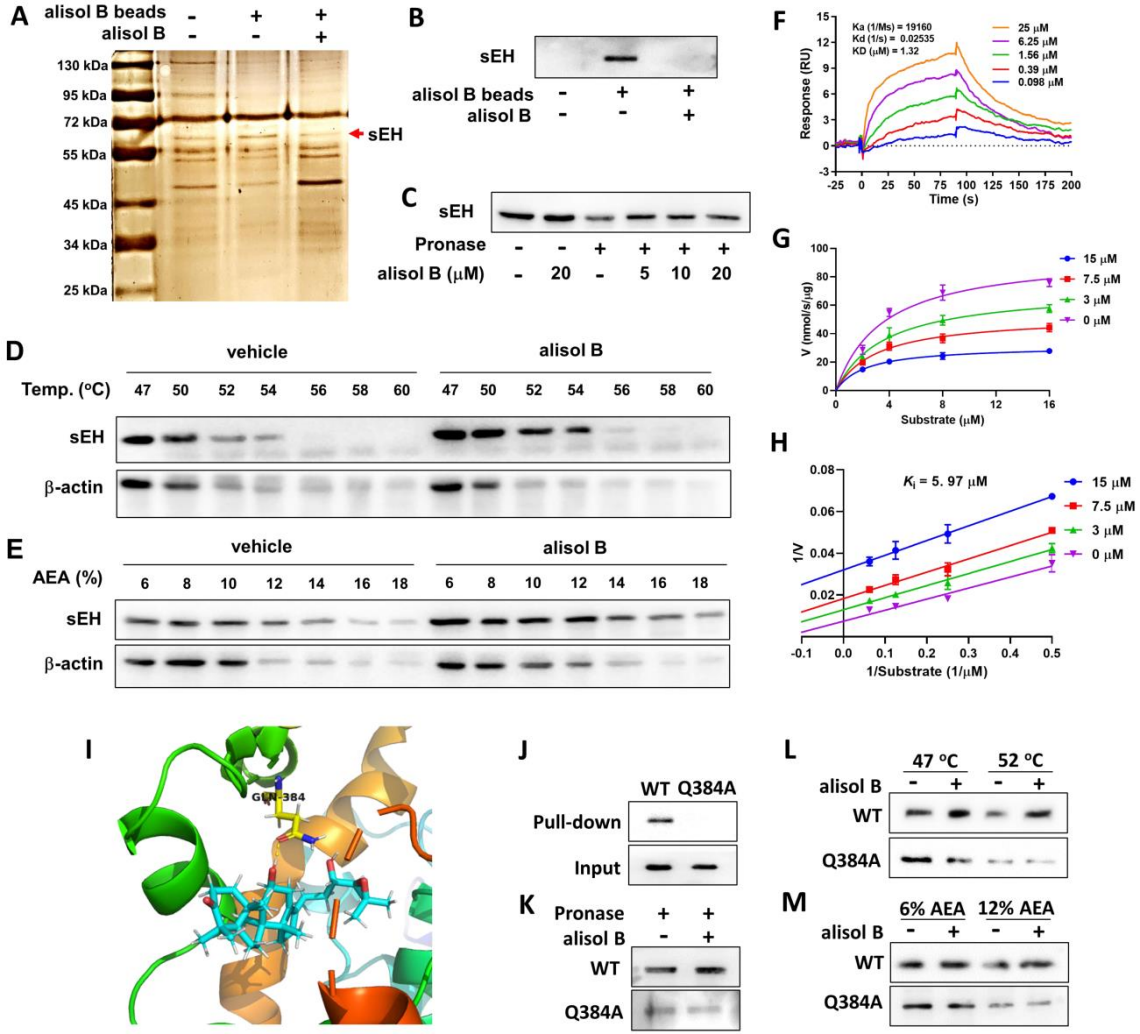
** sEH is a direct target of alisol B. (A)** Identification of cellular target of alisol B using pull-down technology coupled with LC-MS/MS. HEK293 lysate was incubated with control beads or alisol B beads. The binding proteins were detected by silver staining and LC-MS/MS analysis; **(B)** The binding proteins were detected by Western blot; **(C)** HEK293 lysate was incubated with alisol B in the presence or absence of pronase (5 µg/mL); **(D)** HEK293 lysate were exposed to alisol B (50 µM) or vehicle followed by a cellular thermal shift assay; **(E)** HEK293 lysate were exposed to alisol B (50 µM) or vehicle followed by a SIP assay; (**F**) SPR plot of alisol B with sEH; **(G)** Michealis-Menten plot of alisol B against sEH; **(H)** Lineweaver-Burk plot of alisol B against sEH; **(I)** The interaction of alisol B with sEH analyzed by molecular dynamics; **(J-M)** Gln384Ala mutation abolished the binding of alisol B with sEH.

In order to identify the direct cellular targets of alisol B, its epoxyactivated conjugated Sepharose beads were performed as previously described 52, and used as a small-molecule affinity reagent to capture its binding proteins. As described in **Figure 5A**, a protein band at ~63 kDa was identified as soluble epoxide hydrolase (sEH) by LC-MS/MS analysis, which was further supported by Western blot (**Figure 5B&S3**). CETSA, SIP, and DARTS were also used to monitor drug target engagement. The results of CETSA, SIP, and DARTS showed that alisol B alleviated effects of temperature, organic solvent, and pronase on the stability of sEH (**Figure 5D&E**), revealing the direct interaction between sEH and alisol B. The SPR technique was used to analyze the affinity of alisol B and sEH, which demonstrated their equilibrium dissociation constant (*K*_D_ = 1.32 μM,** Figure 5F**). In addition, the inhibition kinetic result revealed its inhibition constant (*K*_i_) value of 5.97 μM (**Figure 5G&H**). The aforementioned results suggested the direct binding of alisol B to sEH.

In order to understand the interaction of alisol B and sEH, molecular dynamics stimulation was used. As described in **Figure 5I&S4**, alisol B could bind to sEH via the hydrogen bond interaction with Gln384 in the catalytic cavity of sEH, therefore Gln384 was mutated into Gln384Ala in order to confirm the role of Gln384. It is worth noting that Gln384Ala abolished the binding of alisol B with sEH (**Figure 5J**), demonstrating that alisol B interacted with amino acid residue Gln384 of sEH. Similarly, CETSA, SIP, and DARTS assays with Gln384Ala mutated sEH further supported our findings (**Figure 5K-M**).

### Alisol B suppressed the sEH activity to stabilize the level of EETs in Cis-induced AKI, further the inhibition of GSK3β

**Figure 6:**
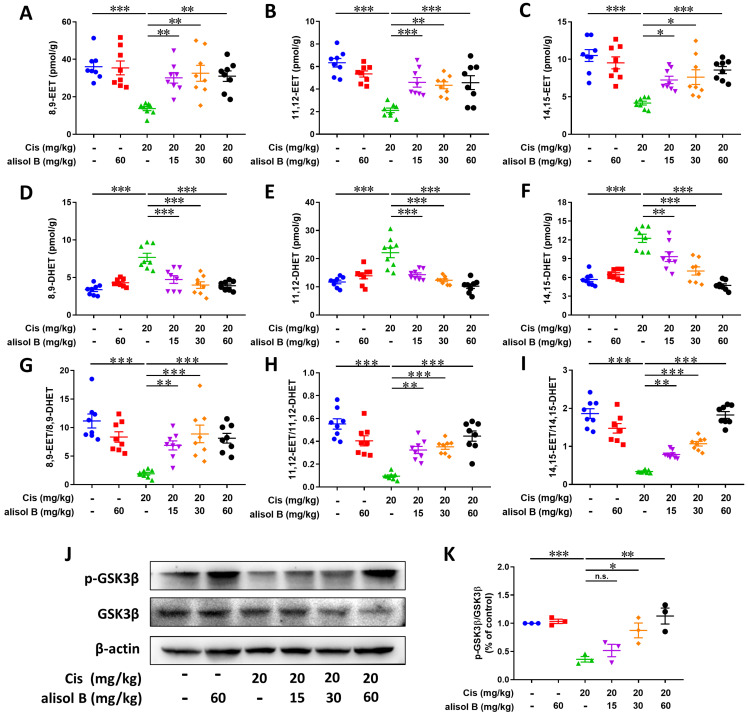
** Alisol B inhibited sEH activity in the kidneys from mice with Cis-induced AKI.** (**A-C**) Levels of EETs regulated by alisol B in the mice with Cis-induced AKI, 8,9-EET (**A**), 11,12-EET (**B**), and 14,15-EET (**C**); data were presented as mean ± SEM, n=8, *p<0.05, ** p<0.01, ***p<0.001; (**D-F**) Levels of DHETs regulated by alisol B in the mice with Cis-induced AKI, 8,9-DHET (**D**), 11,12-DHET (**E**), and 14,15-DHET (**F**); data were presented as mean ± SEM, n=8, *p<0.05, ** p<0.01, ***p<0.001; (**G-I**) The ratio of EETs and DHETs regulated by alisol B in the mice with Cis-induced AKI, 8,9-EET/8,9-DHET (**G**), 11,12-EET/11,12-DHET (**H**), and 14,15-EET/14,15-DHET (**I**); data were presented as mean ± SEM, n=8, *p<0.05, **p<0.01, ***p<0.001; (**J**) Western blot demonstrating the effect of alisol B on protein levels of GSK-3β after Cis challenge; (**K**) Quantitative analysis for the protein levels in (J); data were presented as mean ± SEM, n=3, *p<0.05, ** p<0.01, ***p<0.001, n.s.= no significance.

The result of Sepharose beads demonstrated that the sEH served as a direct cellular target of alisol B, and it is involved in the hydrolytic metabolism of natural epoxides, such as EpFAs (e.g. EETs), to form the corresponding vicinal diol, such as DHETs 58, therefore, LC-MS/MS was applied for analyzing the effect of alisol B on the sEH activity and its metabolites in Cis-induced mice. As shown in** Figure 6A-F**, Cis exposure reduced levels of 8,9-EET, 11,12-EET, and 14,15-EET, and increased levels of 8,9-DHET, 11,12-DHET, and 14,15-DHET, while the reverse results were observed after administration of alisol B (15, 30, and 60 mg/kg). The ratio of EETs and DHETs suggested that alisol B suppressed the sEH activity *in vivo* (**Figure 6G-I**).

Endogenous EETs can regulate the activity of glycogen synthase kinase 3beta (GSK3β) that is a serine/threonine kinase in connection with apoptosis, inflammation, and oxidative stress *via* regulating p53, NF-κB, and Nrf2 signaling pathways 59-61. Therefore, we also determined the effect of sEH inhibition by alisol B on GSK3β. As described in **Figure 6J&K**, the decreased protein level of p-GSK3β by Cis-induced AKI was significantly improved by alisol B administration, which suggested that GSK3β served as a downstream key pathway of sEH in Cis-induced AKI.

**Figure 7:**
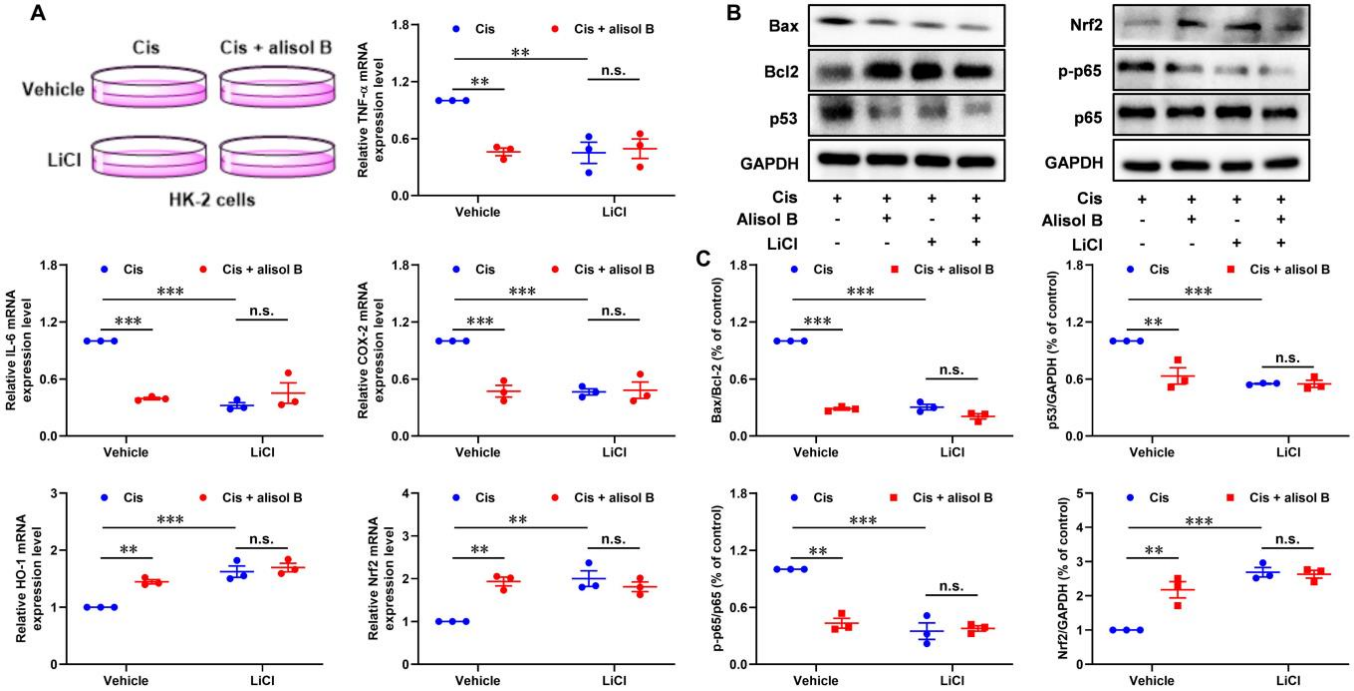
** Inhibition of GSK3β by LiCl abolished the reno-protective effect of alisol B *in vitro*.** (**A**) qPCR analysis showing the mRNA levels of inflammation-related genes, TNF-α, IL-6, COX-2, NQO-1, HO-1, and Nrf2, in Cis-stimulated HK-2 cells with or without alisol B or LiCl; data were presented as mean ± SEM, n=3, ** p<0.01, ***p<0.001, n.s.= no significance. (**B**) Effects of inhibition of GSK3β by LiCl abolished anti-apoptosis, anti-inflammatory, and anti-oxidation effects of alisol B in Cis-stimulated HK-2 cells. (**C**) Quantitative analysis for the protein levels in (**B**); data were presented as mean ± SEM, n=3, ** p<0.01, ***p<0.001, n.s.= no significance.

In order to investigate the role of GSK3β in the treatment of alisol B for Cis-mediated AKI, we performed the GSK3β inhibition experiment in Cis-stimulated HK-2 cells (**Figure 7**). LiCl, a GSK3β inhibitor, decreased the ratio of Bax/Bcl-2 and expression levels of p53 and phosphorylated p65, and enhanced the Nrf2 expression level in Cis-exposed HK-2 cells (**Figure 7B&C**). It is worth noting that alisol B did not display further effects toward Bax/Bcl-2, p53, p-p65, and Nrf2 in Cis-stimulated HK-2 cells after the inhibition of GSK3β by LiCl. Similarly, the inhibition of GSK3β by LiCl abolished effects of alisol B toward mRNA expressions of TNF-α, IL-6, COX-2, HO-1, and Nrf2 in Cis-stimulated HK-2 cells (**Figure 7A**). These results demonstrated that alisol B suppressed the sEH activity to enhance the EETs level, triggering GSK3β-mediated p53, NF-κB, and Nrf2 pathways to attenuate Cis-induced AKI.

### sEH genetic deletion abolished the renal protective effect of alisol B in Cis-induced AKI

**Figure 8:**
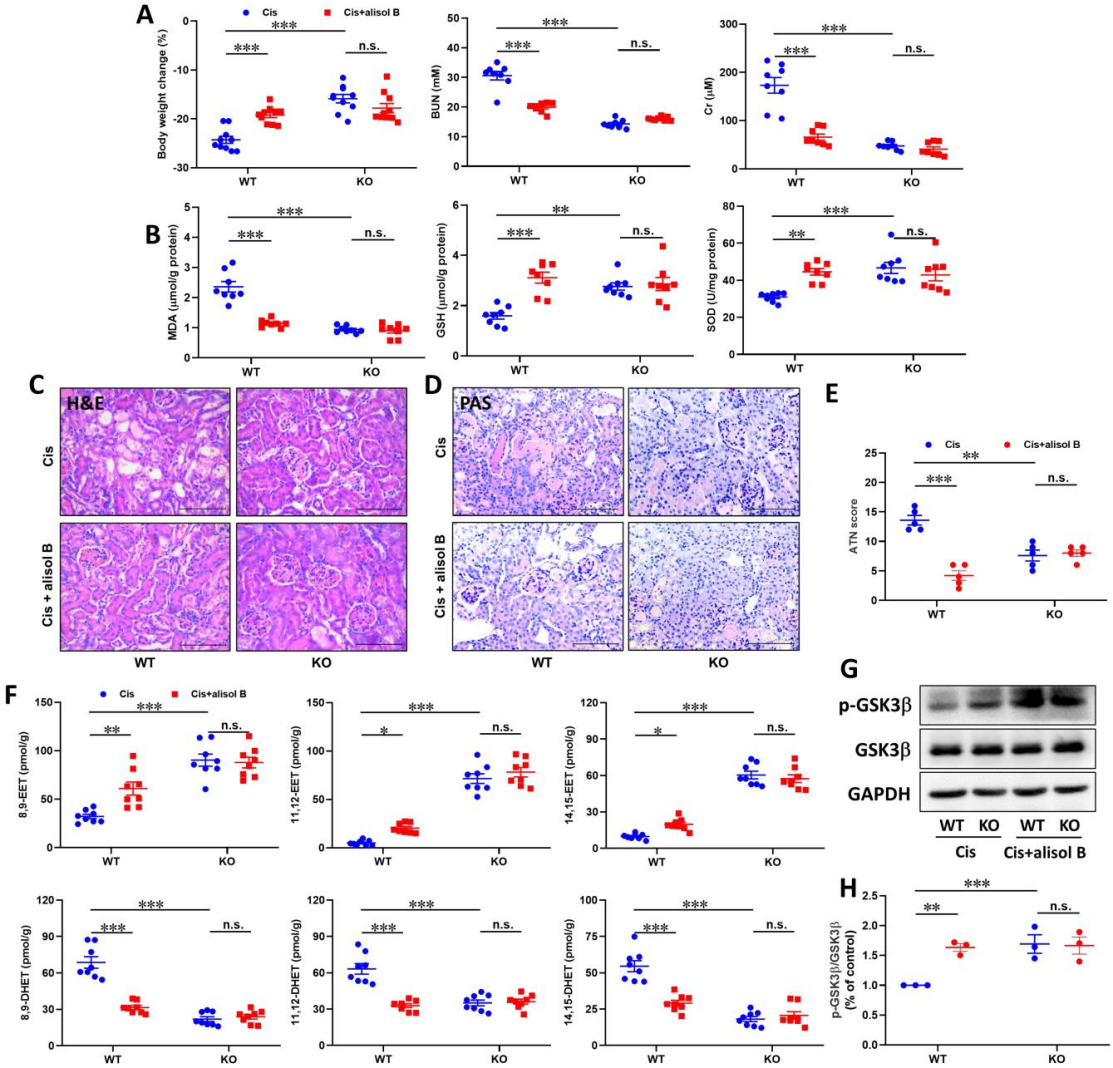
***Ephx2* KO mice abolished the renal protective effect of alisol B. (A)** Measurement of Body weight change, BUN and Cr in WT and sEH^-/-^ mice with Cis-induced AKI treated with alisol B; data were presented as mean ± SEM, n=8-10, *** p<0.001, n.s.= no significance; **(B)** Measurement of renal MDA, GSH, and SOD from WT and sEH^-/-^ mice with Cis-induced AKI treated with alisol B; data were presented as mean ± SEM, n=8, ** p<0.01, ***p<0.001, n.s.= no significance;** (C-D)** Representative images of H&E **(C)** and PAS **(D)** staining in WT and sEH^-/-^ mice with Cis-induced AKI treated with alisol B; **(E)** ATN scoring of histopathological features in different groups of mice; data were presented as mean ± SEM, n=5, ** p<0.01, ***p<0.001; n.s.= no significance;** (F)** Levels of EETs and DHETs regulated by alisol B in WT and sEH^-/-^ mice with Cis-induced AKI treated with alisol B; data were presented as mean ± SEM, n=8, *p<0.05, ** p<0.01, *** p<0.001, n.s.= no significance; **(G)** *Ephx2* KO abolished effects of alisol B on expression levels of p-GSK3β and GSK3β in Cis-induced AKI; **(H)** Quantitative analysis for the protein level in **(G)**; data were presented as mean ± SEM, n=3, ** p<0.01, ***p<0.001, n.s.= no significance.

**Figure 9:**
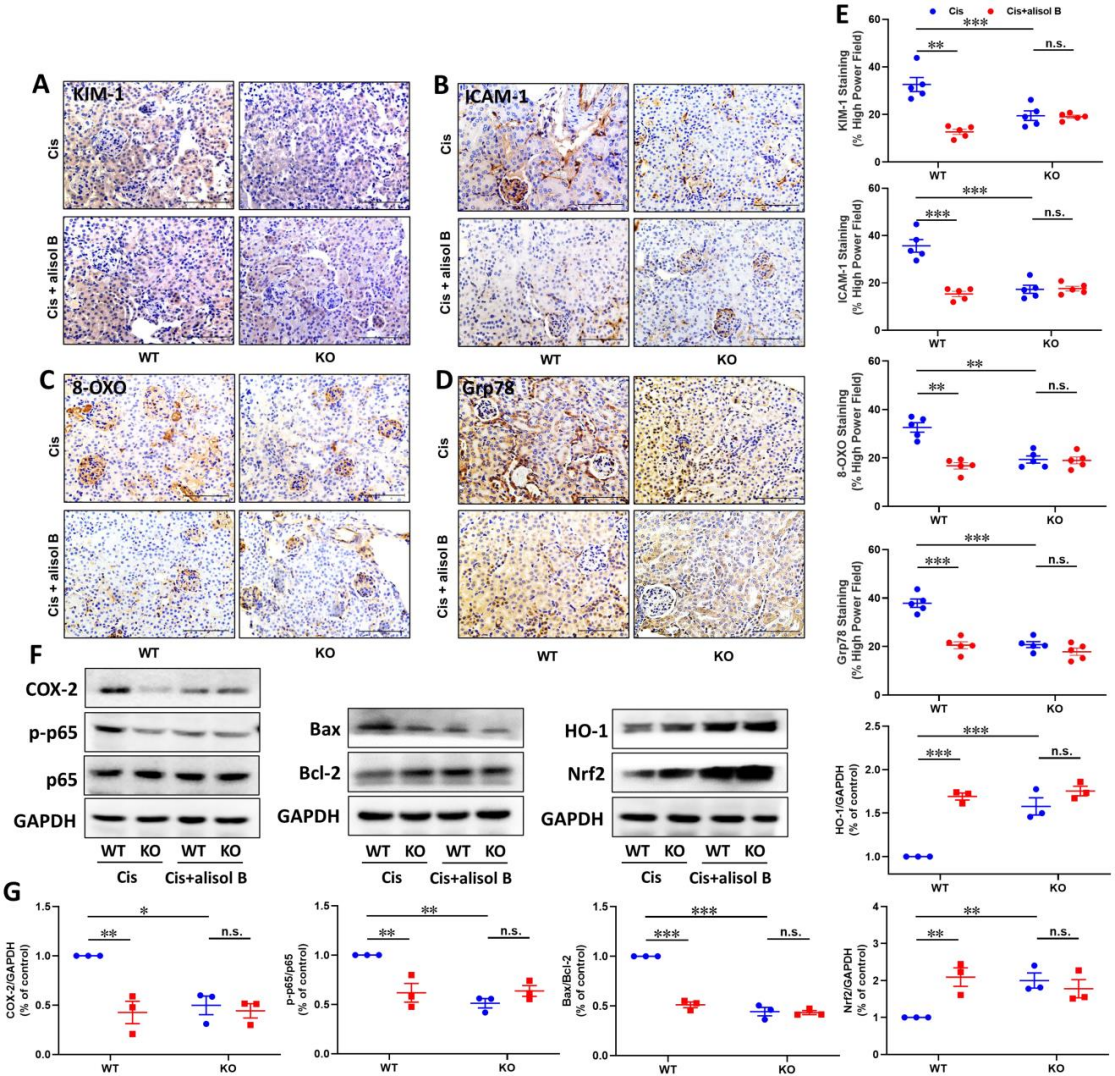
***Ephx2* KO mice abolished the protective effect of alisol B in the apoptosis, inflammation, and oxidative stress. (A-D)** Representative images of immunostaining for KIM-1 (**A**), ICAM-1** (B)**, 8-OXO** (C)**, and Grp78** (D)** in WT and sEH^-/-^ mice with Cis-induced AKI treated with alisol B; **(E)** Quantitative analysis of immunostaining for KIM-1, ICAM-1, 8-OXO, and Grp78; data were presented as mean ± SEM, n=5, ** p<0.01, ***p<0.001; n.s.= no significance; **(F)** *Ephx2* KO abolished effects of alisol B on expression levels of proteins related to inflammation, apoptosis, and oxidative stress in Cis-induced AKI; **(G)** Quantitative analysis for the protein levels in **(F)**; data were presented as mean ± SEM, n=3, *p<0.05, ** p<0.01, ***p<0.001, n.s.= no significance.

To further determine the effect of sEH in the treatment of AKI by alisol B, WT and sEH KO (sEH^-/-^, **Figure S5**) mice were administrated with Cis and alisol B (60 mg/kg). In contrast to WT mice, sEH KO declined renal function and the change of renal morphology induced by Cis (**Figure 8A-E**,** S6, & S7**). Meanwhile, *Ephx2* genetic deletion also over shadowed the protective effect of alisol B (**Figure 8A-E**,** S6, & S7**). The results of EETs and DHETs analyzed by LC-MS/MS illustrated that sEH genetic deletion reversed the reduction of EETs and the increase in DHETs induced by Cis, and promoted the phosphorylation of GSK3β (**Figure 8F-H**). The effect of alisol B was abolished by sEH genetic deletion (**Figure 8F-H**). Furthermore, *Ephx2* genetic deletion suppressed Cis-induced oxidative and ER stresses and inflammation (**Figure 9A-E**), downregulated expression levels of COX-2 and p-p65 and the ratio of Bax/Bcl-2, promoted HO-1 and Nrf2 expressions in Cis-induced AKI mice (**Figure 9F&G**), and abolished the effect of alisol B as well (**Figure 9A-G**). These results demonstrated that sEH genetic deletion abolished the renal protective effect of alisol B, which further supported sEH being a target of alisol B in the AKI.

## Discussion

Since 1978, Cis, a platinum-based alkylating compound, has become an antineoplastic agent to treat solid cancers, including cervical, bladder, and small and non-small cell lung cancers 62. The accumulation of Cis in renal proximal epithelial cells leads to DNA damage, mitochondrial destruction, renal cell apoptosis, inflammatory response, and excessive generation of ROS 63, therefore, its clinical application is greatly limited 64. It is commonly felt in the renal cancer field that lives could be saved if one could increase the dose of Cis at a chemotherapeutic agent if renal damage could be avoided. Although various approaches have been introduced to overcome the side effect of Cis-mediated nephrotoxicity, to date no effective medical treatment strategy has been established for Cis-induced AKI apart from renal replacement therapy. Thus, novel effective therapeutic agents are urgently required to protect patients under Cis-based chemotherapy from renal damage.

As a critical health problem worldwide, AKI causes high mortality and morbidity in the clinic, and increases the risk of CKD and ESRD 65-67. In this study, alisol B, a triterpene from* A. orientale*, was shown to preserve renal function after Cis challenge in mice, and served as a potential protector against Cis-induced AKI. The result of the target fishing experiment demonstrated that alisol B bound to sEH as a direct cellular target through the hydrogen bond with Gln384, which was supported by the results of the inhibition kinetics (*K*_i_ = 5.97 μM) and surface plasmon resonance (*K*_D_ = 1.32 μM). Inhibition of sEH by alisol B stabilized the level of EETs to alleviate renal tubular apoptosis, inflammatory response, and oxidative stress *via* GSK3β-mediated p53, NF-κB, and Nrf2 signaling pathways. Additionally, sEH genetic deletion alleviated the course of Cis-induced AKI and abolished the reno-protective effect of alisol B in Cis-induced AKI mice, revealing that the inhibition of sEH by alisol B to enhance the level of EETs resulted in its renal protective effect through the regulation of apoptosis, oxidative stress, and inflammation. These findings suggested that alisol B could served as a potential candidate for the treatment of AKI.

*A. orientale,* namely “Ze Xie” in Chinese is widely used as a traditional Chinese medicine (TCM) in the treatment of various diseases, such as hyperlipidemia and hypertension 68, 69. Most beneficial effects of *A. orientale* are attributed to its characteristic protostane-type triterpenoids 42, 70-73. Alisol B, one of major protostane-type triterpenes from *A. orientale* 41, 42, has multiple biological effects, including anti-complement, anti-allergy, and anti-inflammation 44, and its 23-acetate displays the protective effect in ischemia-reperfusion-induced AKI 43. Similar to alisol B 23-acetate, alisol B alleviated Cis-induced renal tubule damage, and improved the renal function in Cis-induced AKI mice as well.

The sEH exists in almost all the living organisms. In mammals it is widely distributed in tissues but in particularly high in liver and kidney 29, 30, and mediates the metabolism of epoxides, such as EETs 29. Recently, sEH inhibitors exert anti-inflammation, analgesia, anti-fibrosis, cardioprotection, and renoprotection, because of stabilization of the level of EETs, the epoxide metabolites of arachidonic acid (AA) 7, 30, 37, 74, 75. The expression and activity of sEH have been reported to be associated with kidney diseases 32, and *Ephx2* genetic deletion and chemical inhibition of sEH both attenuate renal diseases 32-34. For example, sEH inhibitors, AUDA and AR9273, significantly attenuated Cis-induced AKI through the decrease of BUN and Cr levels 29, 34, 35. Moreover, *Ephx2* genetic deletion ameliorates the course of Cis-mediated AKI as well 34. Therefore, natural and synthetic sEH inhibitors with favorable pharmacokinetics and pharmacodynamics may act as promising therapeutics for the treatment of Cis-induced AKI. In this study, alisol B was found to bind to sEH *via* inactivating with Gln384 (*K*_D_ = 1.32 μM) and displayed an inhibitory effect with a *K*_i_ value of 5.97 μM, resulting in its reno-protective effect *in vivo*. Furthermore, sEH genetic deletion attenuated the course of Cis-induced AKI, and abolished the protective effect of alisol B.

EETs are bioactive lipid mediators converted from AA by cytochrome P450 2J (CYP2J) or 2C (CYP2C) and the substrates of sEH as well 20. A great body of studies demonstrated that the level of EETs was significantly decreased, and the level of its corresponding diols was remarkable increased in the renal injury 76. Meanwhile, administration of 14,15-EET or improving the level of EETs *via* suppressing sEH reduced cell apoptosis, inflammatory response, and oxidative stress by GSK3β-mediated p53, NF-κB, and Nrf2 signaling pathways in the kidney and central nervous system diseases 51, 59, 61, such as AKI, Parkinson's and Alzheimer's diseases. In addition, administration of EET analogs EET-F01 and EET-A could alleviate Cis-induced nephrotoxicity *via* suppressing apoptosis, inflammation, and oxidative stress 77, 78. Therefore, inhibition of sEH by alisol B to ehance the EETs level is connected with apoptosis, oxidative and ER stresses, and inflammation. The p53 overexpression was observed in Cis-induced AKI mice, and suppressing the p53 expression in Cis-induced AKI ameliorated the apoptosis, suggested the role of p53 in Cis-induced nephrotoxicity 11-14. In this study, alisol B reduced Cis-mediated cell apoptosis by blocking the p53 pathway involved in caspase 3, Bax, Bcl-2, and PARP. Alisol B renoprotection may be also related to decreased inflammation in Cis-treated kidney 64. This speculation is supported by the findings that sEH inhibition by alisol B markedly reduced proinflammatory cytokine expression, including IL-6, TNF-α, ICAM-1, iNOS, COX-2, and MCP-1, which are associated with and probably causative of Cis-induced AKI.

**Figure 10:**
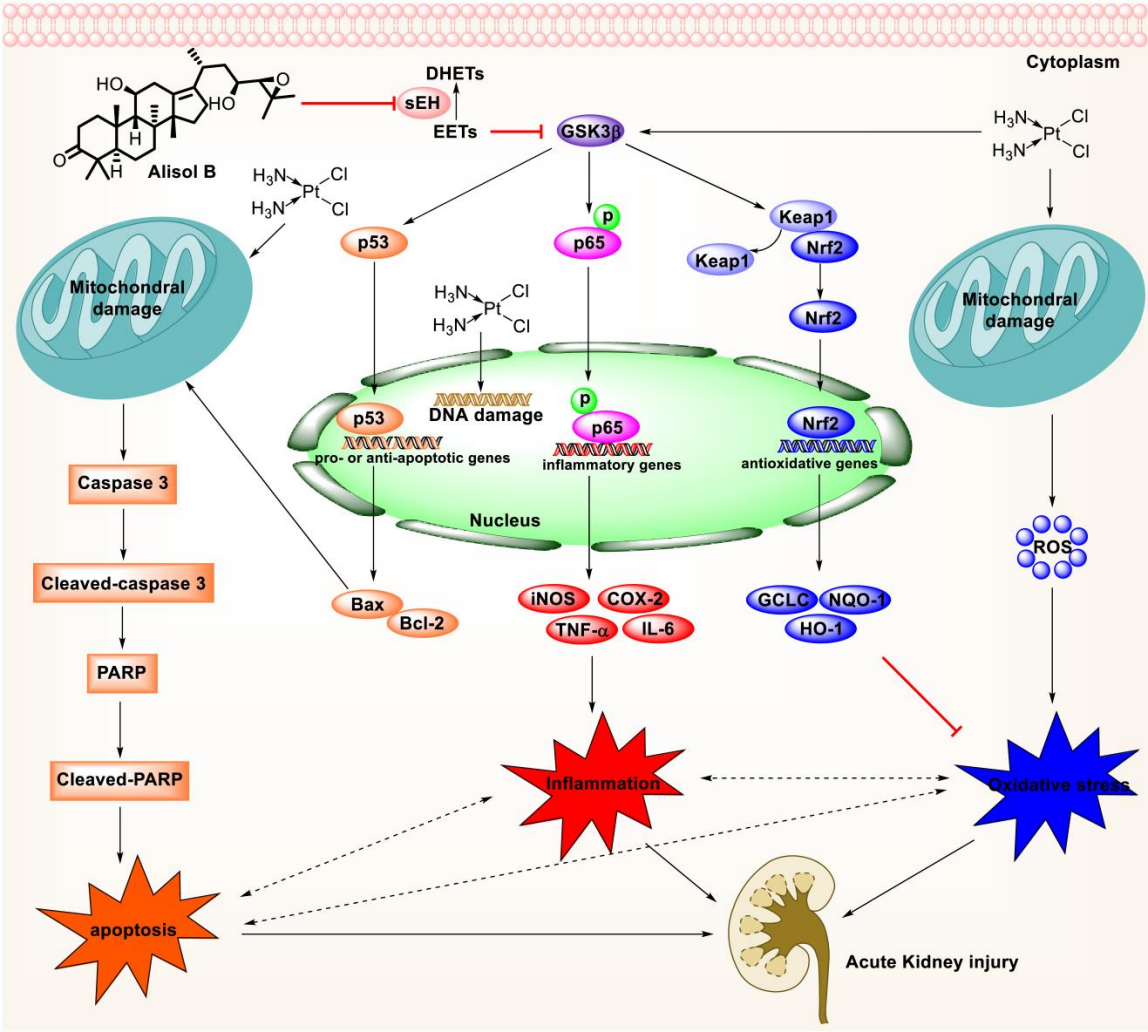
Mechanism of targeting sEH with alisol B in the treatment of Cis-induced AKI.

Furthermore, oxidative and ER stresses have long been considered as important contributors to Cis-induced AKI 2, 79. Oxidative stress promotes histopathological changes through the formation of MDA and the reduction of SOD and GSH levels 80, 81. Inhibition of sEH by alisol B significantly alleviated Cis-induced oxidative stress by reducing MDA level and increasing SOD and GSH levels. Consistently, alisol B treatment significantly reduced oxidative and ER stresses through the Nrf2 activation after Cis treatment. Moreover, the up-regulated level of Grp78, an ER chaperone 82, is inhibited by alisol B. In addition, sEH deficiency attenuated Cis-induced apoptosis, oxidative and ER stresses, and inflammation, and the effect of alisol B in these fields was abolished in Cis-induced sEH KO mice as well (**Figure 10**).

## Conclusion

In summary, alisol B significantly attenuated renal tubular apoptosis, inflammatory response, and oxidative stress in Cis-induced AKI. Furthermore, alisol B targeted sEH to alleviate Cis-induced AKI *via* GSK3β-mediated p53, NF-κB, and Nrf2 signaling pathways, which was supported by the sEH genetic deletion experiment *in vivo*, suggesting that alisol B was a potential candidate to treat drug-induced AKI.

## Supplementary Material

Supplementary figures and tables.

## Abbreviations

AKI: acute kidney injury
BUN: blood urea nitrogen
CETSA: cellular thermal shift assay
CKD: chronic kidney disease
Cis: cisplatin
COX-2: cyclooxygenase-2
Cr: creatinine
DARTS: drug affinity responsive target stability
DHETs: dihydroxyeicosatrienoic acid
EETs: epoxyeicosatrienoic acids
ESRD: end stage renal disease
GSH: glutathione
ICAM-1: intracellular adhesion molecule-1
IL-6: interleukin-6
iNOS: inducible nitric oxide synthase
KO: knockout
MCP-1: monocyte chemoattractant protein-1
MDA: malonyldiadehyde
NF-κB: nuclear factor-kappa B
PAS: periodic acid-Schiff's
PVDF: polyvinylidene difluoride
sEH: soluble epoxide hydrolase
SIP: solvent-induced protein precipitation
SOD: superoxide dismutase
SPR: surface plasmon resonance
TNF-α: tumor necrosis factor-alpha
