# Supplementary material for: Direct targeting of sEH with alisol B alleviated the apoptosis, inflammation, and oxidative stress in cisplatin-induced acute kidney injury
Source: Int J Biol Sci. 2023 Jan 1;19(1):294–310. doi: 10.7150/ijbs.78097 (PMC9760444; doi:10.7150/ijbs.78097)
Supplement: Supplementary file 1 — Supplementary figures and tables. [file ijbsv19p0294s1.pdf]

Direct targeting of sEH with alisol B alleviated the apoptosis, inflammation, and oxidative stress in cisplatin-induced acute kidney injury

Juan Zhang[1,2,+], Zhi-Lin Luan[1,+], Xiao-Kui Huo[1,+], Min Zhang[1], Christophe Morisseau[3], Cheng-Peng Sun[1,*], Bruce D. Hammock[3,*], Xiao-Chi Ma[1,*]

[1]College of Pharmacy, Second Affiliated Hospital, Dalian Medical University, Dalian 116044, People's Republic of China.
[2]School of Pharmaceutical Sciences, Health Science Center, Shenzhen University, Shenzhen 518061, China
[3]Department of Entomology and Nematology, UC Davis Comprehensive Cancer Center, University of California, Davis, CA 95616, United States

[+]These authors contributed equally to this work.

Table S1. Primers used for real-time PCR analysis

Gene	Sequence (5’ to 3’)	
ICAM-1	Forward	GTGATGCTCAGGTATCCATCCA
	Reverse	CACAGTTCTCAAAGCACAGCG
MCP-1	Forward	TTAAAAACCTGGATCGGAACCAA
	Reverse	GCATTAGCTTCAGATTTACGGGT
IL-6	Forward	TAGTCCTTCCTACCCCAATTTCC
	Reverse	TTGGTCCTTAGCCACTCCTTC
TNF-α	Forward	CCCTCACACTCAGATCATCTTCT
	Reverse	GCTACGACGTGGGCTACAG
Nrf2	Forward	CTTTAGTCAGCGACAGAAGGAC
	Reverse	AGGCATCTTGTTTGGGAATGTG
Keap1	Forward	TGCCCCTGTGGTCAAAGTG
	Reverse	GGTTCGGTTACCGTCCTGC
β-actin	Forward	AGCCATGTACGTAGCCATCC
	Reverse	GCTGTGGTGGTGAAGCTGTA

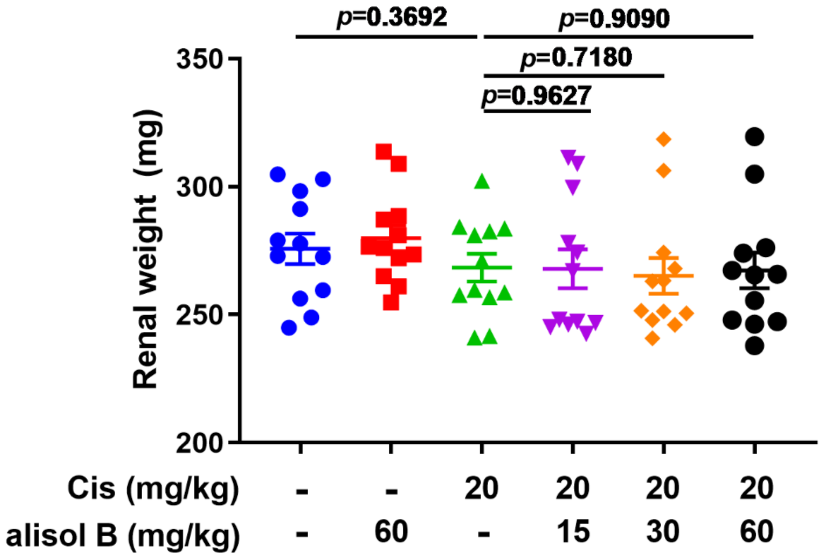

Figure S1. Effects of alisol B against renal weight in WT mice, data were presented as mean ± SEM, n=12.

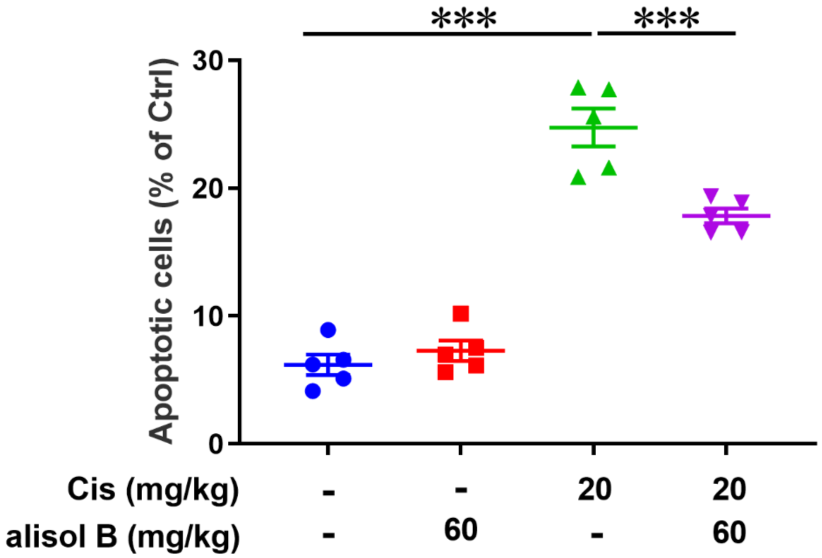

Figure S2. Effects of alisol B against renal weight in WT mice, data were presented as mean ± SEM, n=5, ***p<0.001, n.s.= no significance.

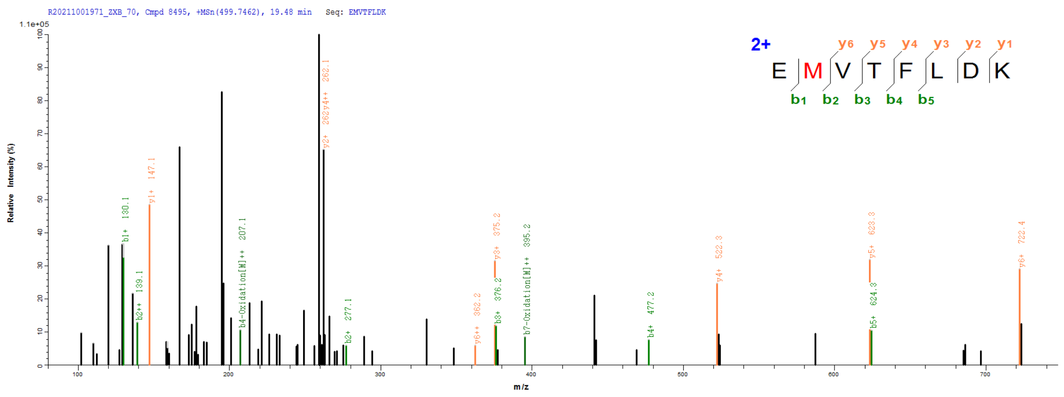

Figure S3. Representative MS/MS spectrum of sEH peptide.

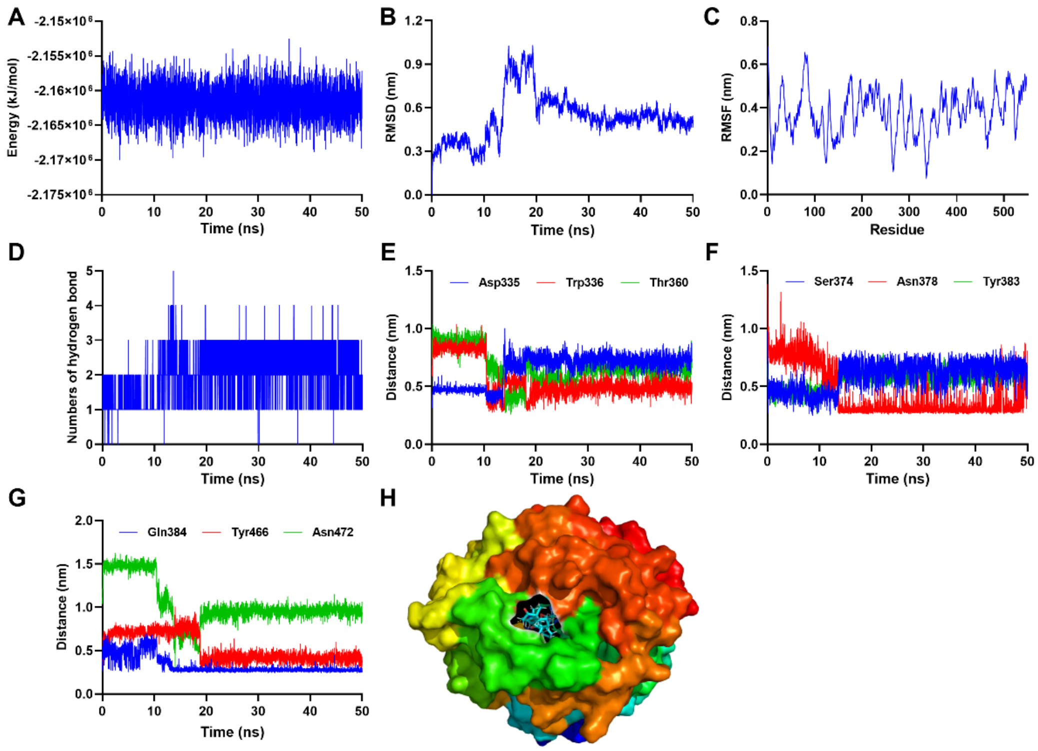

Figure S4. The potential energy (A), RMSD (B), RMSF (C), and hydrogen bond number (D) of alisol B with sEH (PDB: 4OCZ) in the 50 ns of MD. (E-G) The distance of alisol B with amino acid residues Asp335, Trp336, Thr360, Ser374, Asn378, Tyr383, Gln384, Tyr466, and Asn472. (H) The cavity of sEH in the presence of alisol B at 50th ns of MD.

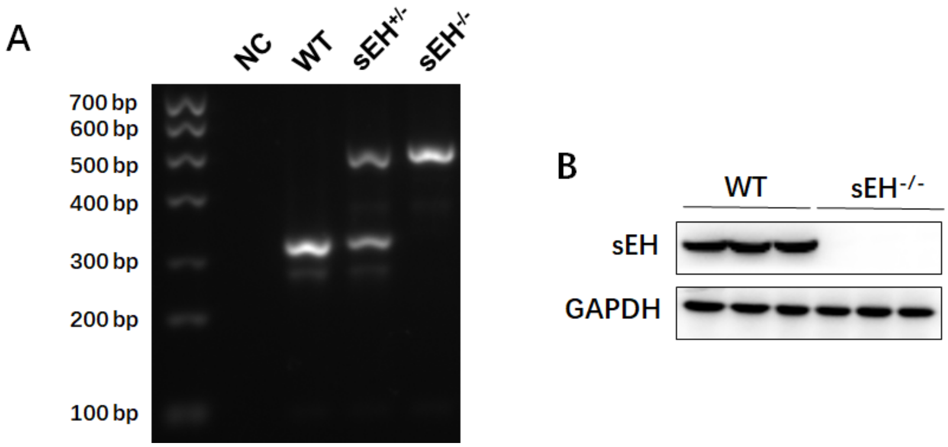

Figure S5. Genotyping and confirmation of sEH knockout mice. (A) Genotyping of $sEH^{-/-}$ mice. The DNA extracted from mouse tails were used for genotyping and amplified by PCR with three primers: 1) F1-5'- TAA TCC AGC AGC TCT CAT GTC ATC -3'; 2) F2-5'- GAA TCA AAC CAT CCA CCC TCT CT -3'; and 3) R-5'-ATT TCT GCC AGA TGT TTT GAG TAC C-3'. The PCR products from wild-type (WT), heterozygote ($sEH^{+/-}$) and homozygote ($sEH^{-/-}$) include only the 322 bp fragment, both the 322 bp and 529 bp fragments, and only the 529 bp fragment, respectively; (B) Western blotting demonstrating that no signal was observed in renal tissues from $sEH^{-/-}$ mice.

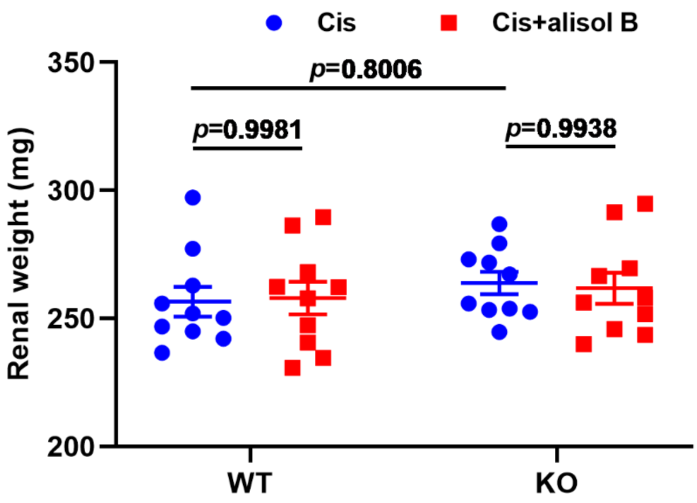

Figure S6. Effects of alisol B against renal weight in WT and *Ephx2* KO mice, data were presented as mean ± SEM, n=10.

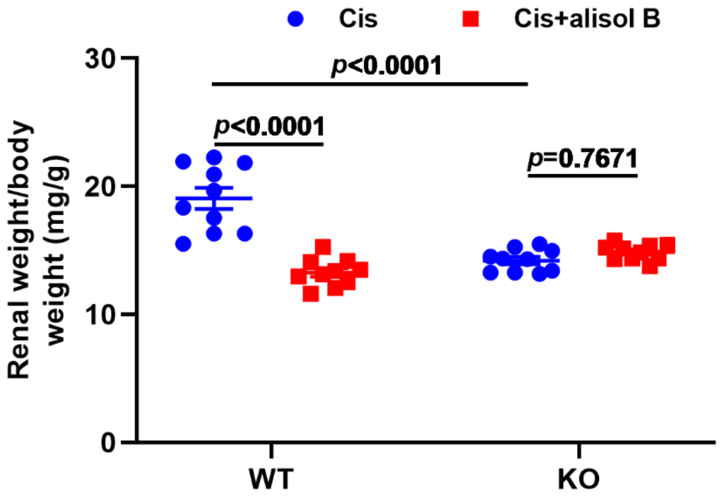

Figure S7. The ratio of renal weight versus body weight of wild-type (WT) and sEH knockout mice with cisplatin-induced AKI after alisol B treatment. Data were presented as mean ± SEM, n=10 per group.

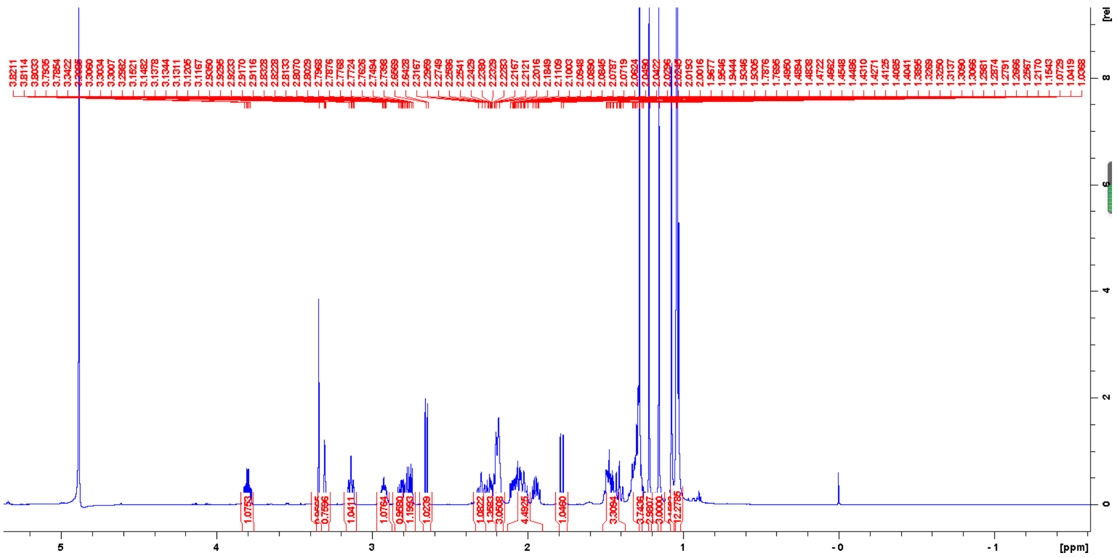

Figure S8. The ^{1}H NMR (CD_3OD, 600 MHz) spectrum of alisol B

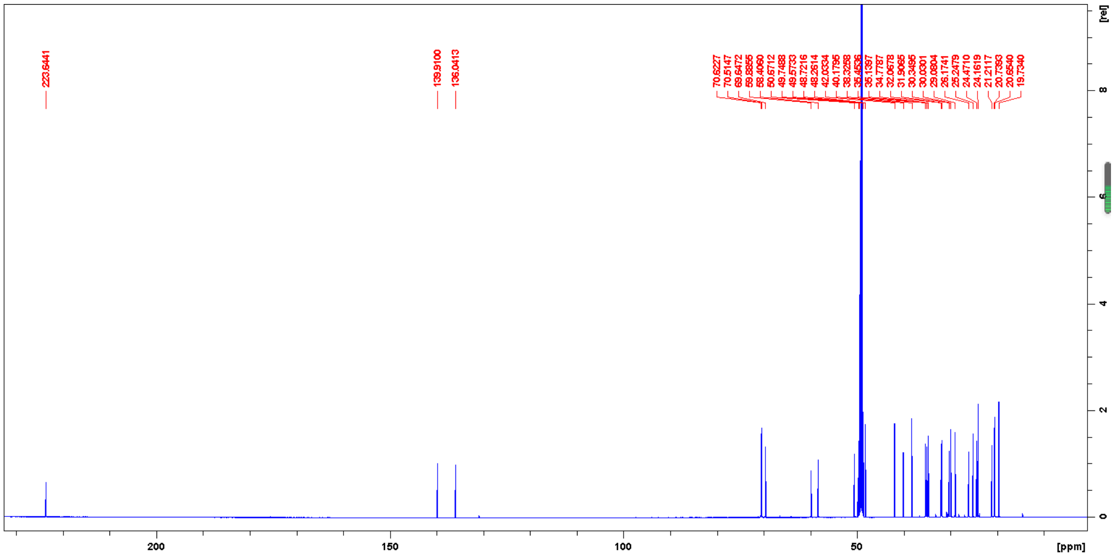

Figure S9. The ^{13}C NMR (CD_3OD, 150 MHz) spectrum of alisol B
